# Nodular fasciitis of the face: A case report

**DOI:** 10.1016/j.ijscr.2019.07.003

**Published:** 2019-07-12

**Authors:** Shems Al-Hayder, Mads Warnecke, Jørgen Hesselfeldt-Nielsen

**Affiliations:** a Department of Plastic Surgery and Breast Surgery, Zealand University Hospital, Roskilde, Sygehusvej 10, 4000 Roskilde, Denmark; b Department of Pathology, Zealand University Hospital, Roskilde, Sygehusvej 10, 4000 Roskilde, Denmark

**Keywords:** Nodular fasciitis, Benign neoplasm, Face, Case report

## Abstract

**Introduction:**

Nodular fasciitis is a benign, self-limited myofibroblastic proliferative lesion of unclear aetiology. It is often misdiagnosed as a malignant tumour due to its rapid growth, high cellularity, and increased mitotic activity.

**Presentation of case:**

We present a case of nodular fasciitis occurring in the medial canthus of a 64-year-old, otherwise healthy, woman. The patient complained of rapidly enlarging asymptomatic subcutaneous nodule over a period of 6 months. The tumour was firm, smooth, and indolent with limited mobility. Fine-needle aspiration was inconclusive, and the CT scan showed a well-defined nodular lesion in the soft tissue without bone destruction. A local excision was performed, and the histopathological findings were consistent with nodular fasciitis.

**Discussion:**

Although rare, nodular fasciitis is the most common pseudosarcoma of soft tissues. Local excision is recommended; however, the tumour often regresses spontaneously, and recurrence is exceedingly rare.

**Conclusion:**

Awareness of nodular fasciitis and its benign nature is essential to avoid misdiagnosis and subsequent inappropriate aggressive treatment of the patient.

## Highlights

• Nodular fasciitis is a benign self-limited lesion.
• It is can misdiagnosed as a sarcoma.
• Local excision is the preferred treatment.
• Spontaneous regression is common.
• Recurrence is rare.

## 1 Introduction

Nodular fasciitis is a benign myofibroblastic proliferative lesion usually affecting subcutis, muscles, and fascia. The process was first described in 1955 by Konwaler et al and was initially named subcutaneous pseudosarcomatous fibromatosis [1]. Although rare, nodular fasciitis is the most common pseudosarcoma of soft tissues [2]. However, it is often misdiagnosed as a malignant tumour due to its rapid growth, infiltrative growth pattern, high cellularity, and increased mitotic activity [[3], [4], [5]].

We present a case of nodular fasciitis occurring in the face to raise awareness of nodular fasciitis as a differential diagnosis in rapidly growing solitary tumours.

## 2 Presentation of case

**Fig. 1:**
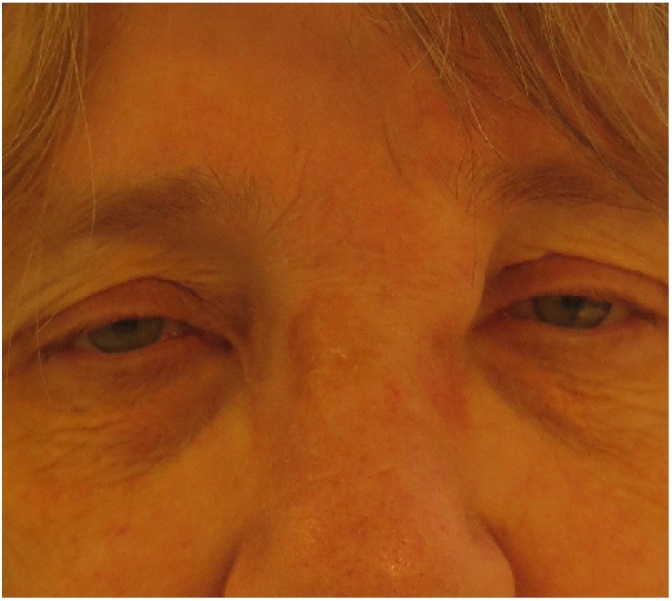
A firm, smooth, and indolent tumour of 20 × 15 mm with limited mobility and no evidence of skin involvement in the left medial canthus.

**Fig. 2:**
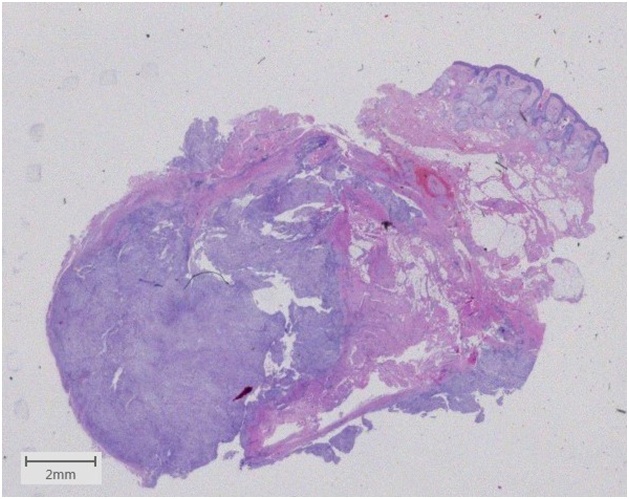
Low magnification of our case showing a well-circumscribed subcutaneous tumour. Dermis and epidermis are shown at the top right. (Hematoxylin and eosin, Slidescanner overview).

**Fig. 3:**
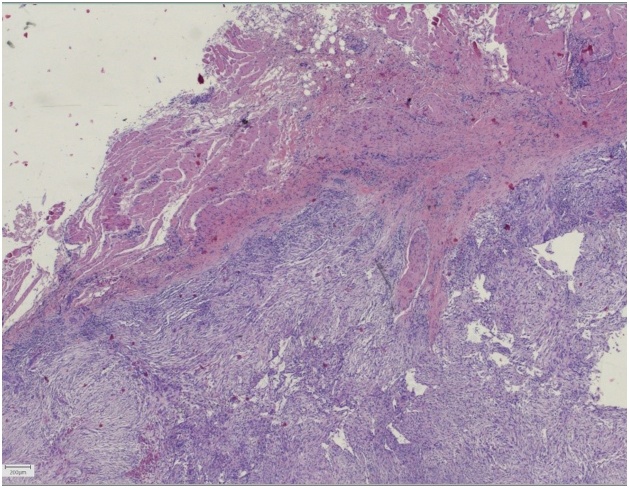
Central part of the tumour with a proliferation of spindle cells, tissue-culture like growth pattern, and close relation to skeletal muscle (top left). (Hematoxylin and eosin, x2,5).

**Fig. 4:**
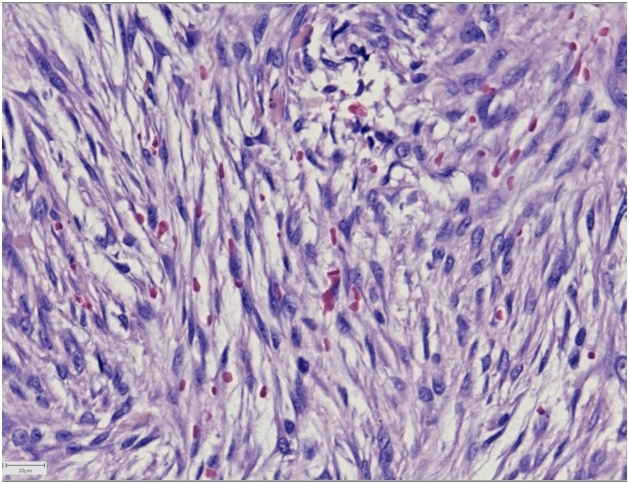
Tumour consisting of a proliferation of myofibroblastic spindle cells within a myxoid stroma with extravasation of erythrocytes (Hematoxylin and eosin, x40).

**Fig. 5:**
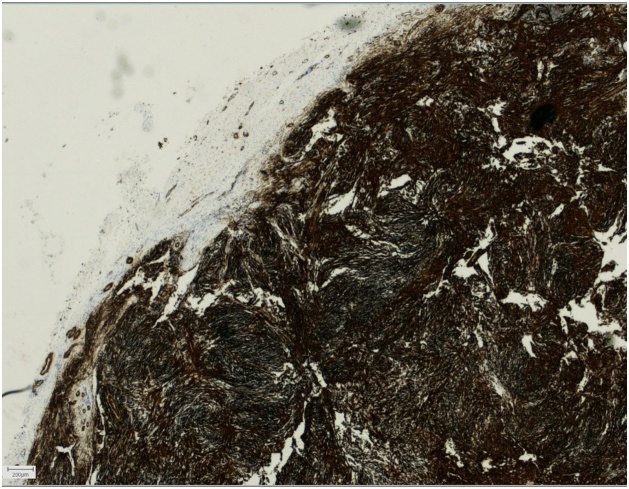
Immunohistochemistry with strong positive reaction for smooth muscle actin (SMA, x25).

A 64-year-old, otherwise healthy, female presented with a 6-month history of a rapidly enlarging asymptomatic tumour in the left medial canthus. There was no history of trauma. A fine-needle aspiration performed by an otolaryngologist was inconclusive. A contrast-enhanced CT scan showed a well-defined nodular lesion in the soft tissue measuring 16 × 12 mm without bone destruction or remodeling. Physical examination in our outpatient department revealed a firm, smooth, indolent process of 20 × 15 mm with limited mobility and no evidence of skin involvement. An excisional biopsy was performed, and macroscopically the lesion was round, greyish-white, unencapsulated and well-circumscribed. Histopathologic examination showed a subcutaneous tumour profoundly in close relation with sparse skeletal muscle. It consisted of a cellular proliferation of myofibroblastic spindle cells with a tissue-culture like growth pattern. The background was myxoid with extravasated red blood cells and few lymphocytes. The cells had small distinct nucleoli without significant atypia. Scattered mitoses were observed, but there were no atypical forms. Immunohistochemical staining was positive for smooth muscle actin while negative for S-100, CD 34, desmin, epithelial membrane antigen, and cytokeratin AE1/AE3 staining. The histopathological findings were found to be consistent with nodular fasciitis (Fig. 1, Fig. 2, Fig. 3, Fig. 4, Fig. 5).

## 3 Discussion

Nodular fasciitis is a benign and self-limiting pseudosarcomatous tumour composed of a vascular and fibroblastic proliferation. It has traditionally been considered as a reactive lesion rather than a true neoplasm. The pathogenesis of nodular fasciitis is not entirely known. Trauma has been implicated as a triggering factor [3,[6], [7], [8]]. However, only a small amount of cases is associated with a history of previous trauma [9,10]. Recently, studies have identified a rearrangement of the ubiquitin-specific protease 6 (USP6) gene as a recurrent and specific finding leading to the increased acceptance that nodular fasciitis represents a clonal neoplastic proliferation [[11], [12], [13]].

Clinically, Nodular fasciitis usually presents as a solitary rapidly growing tender subcutaneous nodule of a few weeks duration and rarely exceeding 2–3 cm in diameter. It occurs mainly during the third to sixth decade of life with men and women equally affected [6,[14], [15], [16]]. The most common location is the upper extremity, particularly the forearm, followed by the trunk, lower extremity, and the head and neck region [3].

Histologically, nodular fasciitis is characterized by a proliferation of spindle cells arranged haphazardly in a myxoid stroma accompanied by a network of capillaries and extravasated erythrocytes. The cells may display an abundance of mitotic figures, however atypia is not observed [17,18]. Nodular fasciitis is classified into three subtypes depending on the relationship with the fascia: subcutaneous, intramuscular, and fascial. The subcutaneous form is the most frequent subtype [9].

Immunohistochemistry is useful to obtain an accurate diagnosis. The characteristic profile is positivity for smooth muscle actin, vimentin, and occasionally focally for desmin, whereas S-100, cytokeratin, and CD34 are not expressed [5,18,19].

Consideration of clinical differential diagnosis includes lipoma, fibromatosis, dermatofibroma, neuroma, neurofibroma, myxoma, benign cyst, benign and malignant fibrous histiocytoma, dermatofibrosarcoma protuberans, fibrosarcoma, leiomyosarcoma, spindle cell carcinoma and melanoma [5,10,17,18].

Local excision of the lesion is the preferred method of treatment, although partial excision may be sufficient as residual nodular fasciitis may subsequently regress by scarring. Even conservative treatment and clinical follow-up may be a good option as many tumours regress spontaneously. Recurrence of nodular fasciitis has been documented, however, it is so rare that recurrence of a lesion classified as nodular fasciitis should lead to a critically review of the original diagnosis as reappraisal often reveals a malignant disease [3,5,10,19].

## 4 Conclusion

Diagnostic issues with nodular fasciitis are common. The lesion may easily be mistaken for a malignant tumour due to its rapid growth and histopathologic similarities. Awareness of nodular fasciitis and its benign nature among clinicians is essential to avoid misdiagnosis and subsequent inappropriate aggressive treatment of the patient.

This work has been reported in line with the SCARE criteria [20].

## Sources of funding

The authors have nothing to declare.

## Ethical approval

The case report is exempt from ethical approval in our institution.

## Consent

Written informed consent was obtained from the patient for publication of this case report and accompanying images. A copy of the written consent is available for review by the Editor-in-Chief of this journal on request.

## Author’s contribution

Shems Al-Hayder: study concept and design, data collection, data interpretation, writing the paper.

Mads Warnecke: Study concept, data collection, data interpretation, writing the paper.

Jørgen Hesselfeldt-Nielsen: data interpretation and writing the paper.

## Registration of research studies

N/A.

## Guarantor

Shems Al-Hayder.

## Provenance and peer review

Not commissioned, internally peer-reviewed.

## Declaration of Competing Interest

The authors have nothing to declare.
